# Beneficial effect of ezetimibe-atorvastatin combination therapy in patients with a mutation in ABCG5 or ABCG8 gene

**DOI:** 10.1186/s12944-019-1183-4

**Published:** 2020-01-04

**Authors:** Hayato Tada, Hirofumi Okada, Akihiro Nomura, Masayuki Takamura, Masa-aki Kawashiri

**Affiliations:** Department of Cardiovascular Medicine, Kanazawa University Graduate School of Medical Sciences, 13-1 Takara-machi, Kanazawa, 920-8641 Japan

**Keywords:** ABCG5, ABCG8, LDL cholesterol, Ezetimibe, Sitosterolemia

## Abstract

**Background:**

Use of ezetimibe on top of statin therapy has been shown to be effective to reduce LDL cholesterol level in hypercholesterolemic patients. However, little is known regarding the individual variety of the effectiveness of ezetimibe. We hypothesized that hypercholesterolemic patients with a mutation in *ABCG5* or *ABCG8* gene exhibit better response to ezetimibe than those without, based on the fact that ezetimibe is hyper-effective for in patients with sitosterolemia caused by *ABCG5* or *ABCG8* genetic mutations.

**Methods:**

Electronical medical record were reviewed in a total of 321 hypercholesterolemic patients (baseline LDL cholesterol = 192 ± 46 mg/dl) prescribed ezetimibe 10 mg daily on top of atorvastatin 10 mg daily who had undergone genetic analysis of *ABCG5* or *ABCG8* gene in our institute since 2006 to 2017. Pathogenicity of the variants were determined using standard variant filtering schema, including minor allele frequency, in silico annotation tools. Patients were divided into 2 groups based on the presence of *ABCG5* or *ABCG8* mutation. We compared the percent reduction of LDL cholesterol as well as the achieved LDL cholesterol levels between these 2 groups.

**Results:**

We found 26 (8%) individuals who exhibit deleterious mutations in *ABCG5* or *ABCG8* gene. Baseline characteristics under the atorvastatin 10 mg therapy were comparable in age, gender, and LDL cholesterol level between 2 groups. Under these conditions, percent reduction of LDL cholesterol in mutation positive group was significantly larger than that of mutation negative group (28 ± 16% vs. 39 ± 21%, *p* < 0.05). As a result, the achieved LDL cholesterol level in mutation positive group was significantly lower than that of mutation negative group (87 ± 29 mg/dl vs. 72 ± 26% mg/dl, *p* < 0.05).

**Conclusion:**

These results suggest that ezetimibe-atorvastatin combination therapy might be more beneficial in hypercholesterolemic patients with a mutation in *ABCG5* or *ABCG8* gene.

## Introduction

LDL cholesterol has been shown as a causal factor for atherosclerotic cardiovascular disease (ASCVD) by epidemiological studies, randomized controlled trials (RCT), and Mendelian randomized studies [1–3]. Accumulated evidence have repeatedly shown the lower, the better story in the case of LDL cholesterol and ASCVD [4]. There are several different types of LDL-lowering strategies; among them, HMG-CoA reductase inhibitors (statins) have been established as the first line for preventive cardiology based on numerous clinical evidence, including efficacy as well as safety. However, there are substantial proportions of individuals whose LDL cholesterol level cannot be reduced adequately even under maximum dose of statins [5, 6]. Under these conditions, several medical therapies, including ezetimibe, proprotein convertase subtilisin/kexin type 9 (*PCSK9*) inhibitors, and a cholesteryl ester transfer protein (*CETP*) inhibitor have been shown to be effective to reduce ASCVD events on top of statins [7–10]. Among those novel therapies, ezetimibe is an unique drug which has been developed based on the basic research revealing that Niemann-Pick C1-Like 1 (*NPC1L1*) is an important transporter which works absorbing sterols, including cholesterol from intestine [11]. It is reasonable to add ezetimibe on top of statins to further reduce LDL cholesterol, since serum cholesterol roughly comes from synthesis from liver by 70%, and from absorption from intestine by 30% [12]. In addition, it has been shown that cholesterol absorption is increased under statins therapies [13]. Several Mendelian randomization study as well as RCT clearly show us beneficial effect of reducing LDL cholesterol by inhibiting NPC1L1 in addition to inhibiting HMG-CoA reductase [14, 15]. However, there are some sub-analyses suggesting that effectiveness of ezetimibe might be somewhat different according to characteristics of patients [16, 17]. Researchers including ourselves have shown the great effectiveness of ezetimibe in the case of sitosterolemia who has double mutations in ATP-binding cassette sub-family G member 5 or 8 (*ABCG5*) or (*ABCG8*) gene [18–21]. Moreover, we have shown that deleterious mutation(s) in *ABCG5* or *ABCG8* gene are contributing to elevation of LDL cholesterol substantially [22]. Those observation could motivate us to investigate whether using ezetimibe on top of statins in the patients with *ABCG5* or *ABCG8* mutation could be more beneficial compared to those without. In this study, we retrospectively investigated our electrical medical record to see this matter.

## Materials and methods

### Study population

**Fig. 1:**
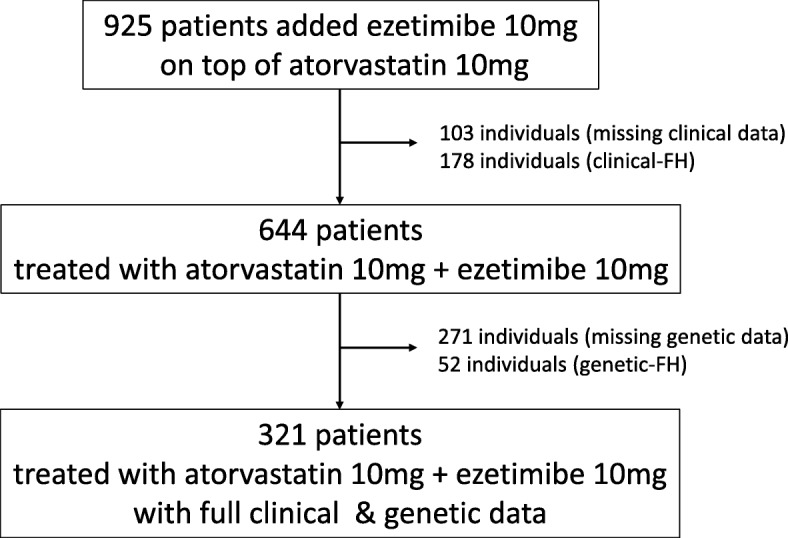
Study flow chart. A group of 925 patients at Kanazawa University Hospital treated using ezetimibe 10 mg/day on top of atorvastatin 10 mg/day between April 2006 to March 2017 were investigated. We excluded 103 patients because of missing clinical data, 178 individuals because their status of clinical FH, 271 individuals lacking genetic analyses on *ABCG5*/*ABCG8* genes, 52 individuals with genetic-FH. Finally, a cohort of 321 participants with a mean age of 51 ± 18 years was included in this retrospective analysis

A group of 925 patients at Kanazawa University Hospital treated using ezetimibe on top of atorvastatin 10 mg/day between April 2006 to March 2017 were screened (Fig. 1). Screening excluded 103 patients for missing clinical data, 178 individuals with clinical FH, 271 individuals lacking genetic analyses on *ABCG5*/*ABCG8* genes, 52 individuals with genetic-FH. Finally, a cohort of 321 participants with a mean age of 51 ± 18 years was included in this retrospective analysis. There were 172 men (52%), and baseline LDL cholesterol level was 192 ± 46 mg/dl.

### Genetic analysis

Genomic DNA was isolated from peripheral white blood cells. DNA was pooled, selected for size, ligated to sequencing adapters, and amplified to enrich for targets that were sequenced using the Kapa DNA Library Preparation. A custom NimbleGen in-solution DNA capture library (Roche NimbleGen Inc., Madison, WI) was designed to capture all coding exons regions of 21 dyslipidemia-related genes with Mendelian inheritance, including *ABCG5* and *ABCG8* genes. Target-enriched products were sequenced using the Illumina MiSeq. The target coverage for each subject was ≥20-fold in ≥98% of all targeted exons. The pathogenicity of the variants were determined by allele frequency, *in-silico* analysis, and Clinvar (https://www.clinicalgenome.org/data-sharing/clinvar) as previously described [22]. In addition, four SNPs validated in assessing polygenic cause of FH in East Asian patients were sequenced. Weighted polygenic scores were calculated based on LDL cholesterol raising alleles and their effect sizes shown in the literature [22]

### Ethical considerations

The study was approved by the Ethics Committee of Kanazawa University and conducted following the ethical standards of the responsible committee on human experimentation (institutional and national) and with the Helsinki Declaration of 1975, as revised in 2008. The study participants gave informed consent for genetic analysis before inclusion.

### Biochemical analysis

Blood samples were collected after overnight fasting, and serum total cholesterol, triglycerides and high-density lipoprotein (HDL) cholesterol were assayed enzymatically with an autoanalyzer (Qualigent, Sekisui Medical, Tokyo, Japan). If triglycerides were < 400 mg/dL, then LDL cholesterol concentration was calculated with the Friedewald equation; if not, then it was determined enzymatically. Baseline was assessed just before the introduction of atorvastatin 10 mg/day, and lipids with atorvastatin 10 mg/day was assessed just before the introduction of ezetimibe 10 mg/day. Lipids with atorvastatin 10 mg/day and ezetimibe 10 mg/day were assessed just after the addition of ezetimibe 10 mg/day (the interval was at least 4 weeks).

### Clinical evaluation

Hypertension was defined as a systolic blood pressure of ≥140 mmHg and a diastolic blood pressure of ≥90 mmHg or the use of antihypertensive medications. Coexisting diabetes was defined as described by the Japan Diabetes Society or the use of diabetes medications.

### Statistical analysis

Categorical variables were reported as percentages and compared with Fisher’s exact test or the chi-square test, whichever was appropriate. Continuous variables with a normal distribution were reported as means ± standard deviation (SD). Variables that were not normally distributed were reported as medians and interquartile range (IQR). Mean values of continuous variables were compared with Student’s *t*-test for independent data; and median values were compared with the nonparametric Wilcoxon Mann–Whitney rank sum test, or the chi-squared test for categorical variables with Fisher’s post-hoc test. In addition, we dichotomized the individuals based on the responsiveness of additional ezetimibe therapy, where responder included individuals whose percent reduction of LDL cholesterol was greater than the median. And we performed multivariate logistic regression analysis to see if *ABCG5* or *ABCG8* genetic mutation status was independently associated with better response. The statistical analysis was conducted with R statistics (https://www.r-project.org). *P*-values of < 0.05 were considered statistically significant.

## Results

### Participant characteristics

The clinical characteristics of the study participants are shown in Table 1. The mean age was 51 ± 18 years, mean baseline LDL cholesterol was 192 mg/dL, mean LDL cholesterol level after the introduction of atorvastatin 10 mg/day was 121 mg/dl, and mean LDL cholesterol under the atorvastatin 10 mg/day and ezetimibe 10 mg/day was 83 mg/dl. Weighted polygenic score in the *ABCG5/ABCG8* mutation-positive group was significantly lower than that in mutation-negative group (Table 1).

**Table 1:** Characteristics of the study subjects

	All	*ABCG5/8* mutations		
Variable	(*n* = 321)	YES (*n* = 26)	NO (*n* = 295)	*p* value
Age (years)	51 ± 18	52 ± 13	51 ± 16	n.s.
Gender (male/female)	172/142	12/14	160/135	n.s.
Hypertension	92 (29%)	7 (27%)	85 (29%)	n.s.
Diabetes	118 (37%)	9 (35%)	109 (37%)	n.s.
Smoking	70 (22%)	5 (19%)	65 (22%)	n.s.
Weighted polygenic score	0.53 ± 0.10	0.46 ± 0.11	0.54 ± 0.10	< 0.05
Baseline LDL-cholesterol (mg/dl)	192 ± 46	194 ± 56	191 ± 41	n.s.
LDL-cholesterol (mg/dl) under atorvastatin 10 mg/day	121 ± 48	121 ± 45	120 ± 34	n.s.
LDL-cholesterol (mg/dl) under atorvastatin 10 mg/day + ezetimibe 10 mg/day	83 ± 30	72 ± 26	87 ± 29	< 0.05

*ABCG5* ATP-binding cassette sub-family G member 5, *ABCG8* ATP-binding cassette sub-family G member 8

### Genotype in ABCG5 or ABCG8 and responses to atorvastatin and ezetimibe

**Fig. 2:**
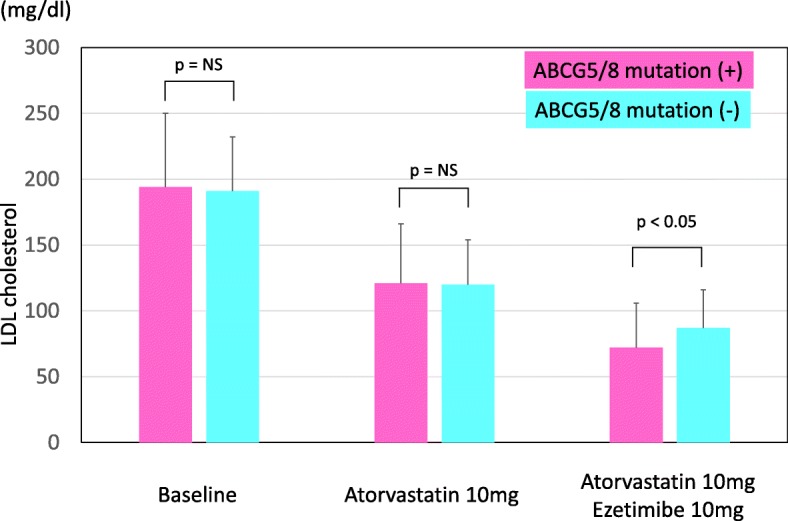
Changes in LDL cholesterol during treatments. Pink bars indicate patients with a ABCG5/8 mutation. Blue bar indicate patients without ABCG5/8 mutations

We identified 10 different mutations in *ABCG5* or *ABCG8* gene in 26 individuals among 321 study subjects (Table 2). Baseline LDL cholesterol levels between 2 groups were not different (Fig. 2). In addition, LDL cholesterol levels obtained under atorvastatin 10 mg/day between 2 groups were not different, too. On the other hand, LDL cholesterol levels obtained under atorvastatin 10 mg/day and ezetimibe 10 mg/day in patients with mutations in *ABCG5* or *ABCG8* gene exhibited significantly lower than those of individuals without any mutations in *ABCG5* or *ABCG8* gene (Table 1, Fig. 2**,** 72 ± 26% mg/dl vs. 87 ± 29 mg/dl, *p* < 0.05).

**Table 2:** Identified mutations

Gene	Nucleotide Change	Mutation Type	Effect on Protein	Number of Patients	ACMG Pathogenicity Criteria
*ABCG5*	c.348C > A	Missense	p.Asn116Lys	2	Supporting (PP1)
*ABCG5*	c.635-1G > A	Splice-cite	NA	2	Very strong (PVS1)
*ABCG5*	c.1166G > A	Missense	p.Arg389His	5	Moderate (PP1/PP4)
*ABCG5*	c.1673_1677delCTTTT	Frameshift	p.Pro558GlnfsTer14	1	Very strong (PVS1)
*ABCG8*	c.55G > C	Missense	p.Asp19His	1	Supporting (PP1)
*ABCG8*	c.1226A > G	Missense	p.Asn409Ile	1	Supporting (PP1)
*ABCG8*	c.1256 T > A	Missense	p.Ile419Asn	6	Moderate (PP1)
*ABCG8*	c.1285A > G	Missense	p.Met429Val	3	Moderate (PP1)
*ABCG8*	c.1763C > A	Missense	p.Ala588Glu	3	Supporting (PP1)
*ABCG8*	c.1798 T > G	Missense	p.Phe600Val	2	Supporting (PP1)

*ABCG5* ATP-binding cassette sub-family G member 5, *ABCG8* ATP-binding cassette sub-family G member 8, *ACMG* American College of Medical Genetics and Genomics

**Fig. 3:**
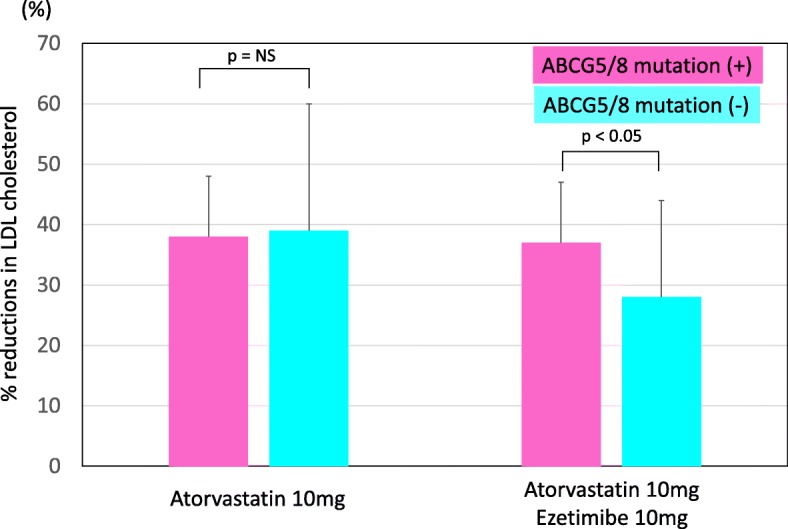
Percent reduction in LDL cholesterol during treatments. Pink bars indicate patients with a ABCG5/8 mutation. Blue bar indicate patients without ABCG5/8 mutations

Under these conditions, percent reduction of LDL cholesterol in mutation positive group was significantly larger than that of mutation negative group (28 ± 16% vs. 39 ± 21%, *p* < 0.05, Fig. 3).

### Factors associated with better response to additional ezetimibe therapy

We found that *ABCG5* or *ABCG8* genetic mutation status was significantly associated with better response (percent reduction in LDL cholesterol was greater than the median), independent of other factors (odds ratio [OR] = 2.29, 95% confidence interval [CI], 1.23–3.32, *p* < 0.05, Table 3). Interestingly, we also found that diabetic status was also associated with better response (OR = 1.46, 95% CI, 1.06–2.00, *p* < 0.05).

**Table 3:** Factors associated with better response to additional ezetimibe therapy

Variable	OR	95% CI	*p* value
Age	1.09	0.87–1.31	0.29
Gender male	0.79	0.22–2.65	0.68
Hypertension	1.32	0.49–2.87	0.28
Diabetes	1.46	1.06–2.00	0.02
Smoking	0.78	0.23–2.02	0.33
Baseline LDL cholesterol (per 10 mg/dl)	1.12	0.78–1.44	0.55
*ABCG5/8* mutation status	2.29	1.23–3.32	0.0034

*OR* odds ratio, *CI* confidence interval, *ABCG5* ATP-binding cassette sub-family G member 5, *ABCG8* ATP-binding cassette sub-family G member 8

## Discussion

In this study, we investigate whether using ezetimibe 10 mg/day on top of atorvastatin 10 mg/day to the patients with *ABCG5* or *ABCG8* mutation could be more beneficial compared to those without. We found that percent reduction of LDL cholesterol in mutation positive group by the use of ezetimibe 10 mg/day on top of atorvastatin 10 mg/day was significantly larger than that of mutation negative group, leading to significant lower achieved LDL cholesterol level in mutation positive group compared with mutation negative group. We also found that *ABCG5* or *ABCG8* genetic mutation status as well as diabetic status was independently associated with better response. These results suggest that ezetimibe-atorvastatin combination therapy might be more beneficial in hypercholesterolemic patients with a mutation in *ABCG5* or *ABCG8* gene.

*ABCG5* or *ABCG8* gene have been shown to be associated with LDL cholesterol level through common genetic variations as well as rare variations leading to sitosterolemia, a recessive Mendelian disorder [21, 23]. Recently, we have shown that there are a portion of individuals with *ABCG5* or *ABCG8* genetic mutations exhibiting extremely high level of LDL cholesterol [24]. Those facts suggest that there are substantial proportions of individuals with hyper LDL cholesterolemia caused by *ABCG5* or *ABCG8* genetic mutation(s). The supposed mechanisms of their hyper LDL cholesterolemia is increased absorption of sterols from intestine as observed in the most extreme cases of sitosterolemia who have double mutations. On the other hand, ezetimibe has been developed to inhibit *NPC1L1*, which play a pivotal role in the absorption of sterols from intestine. And this unique drug has been shown to be quite effective to reduce serum sterols in the cases with sitosterolemia [18–20]. In that sense, it is no surprising to see our results that use of ezetimibe is effective in mutation carriers of *ABCG5* or *ABCG8* gene. However, to the best of our knowledge, there is no prior data exist regarding the effectiveness of ezetimibe, especially, on top of statin therapy among the patients with mutation carriers of *ABCG5* or *ABCG8* gene. In addition, there are several reports showing that clinical benefit of ezetimibe might be different according to patients’ characteristics [16, 17]. Those facts collectively suggest that at least a part of this variability of effectiveness of ezetimibe could be explained by the mutation status of *ABCG5* or *ABCG8* gene. Moreover, such cholesterol absorption markers have been shown as a surrogate for elevated ASCVD risk [25–27]. We believe that ezetimibe should be introduced on top of statins to such patients where cholesterol absorption might be increased, including via genetic backgrounds. In addition, we can estimate if the addition of ezetimibe could be enough for their LDL cholesterol control based on genotype, in the condition where we have options, including PCSK9 inhibitor.

Recent advances of genetic analyses motivate us to establish a precision medicine for particular diseases based on genotypes of an individual [28, 29]. In the field of cardiovascular genetics, researchers have shown that information from rare genetic variants in addition to common genetic variations could be used for their risk stratification at the more advanced level compared with other traditional risk factors [30]. We found that the weighted polygenic score in the *ABCG5/ABCG8* mutation-positive group was significantly lower than that in mutation-negative group. Those results suggest that the etiology of hyper LDL cholesterolemia is somewhat different between those 2 groups. Under these conditions, the effect of ezetimibe 10 mg was different, whereas, that of atorvastatin 10 mg was not different. Based on those facts, investigating the genetic backgrounds including weighted polygenic score as well as rare genetic variations, such as *ABCG5/ABCG8* gene, could lead to their precision medicine. We believe that genetic analyses for *ABCG5/ABCG8* gene are useful not only to identify carriers of sitosterolemia (patients with hyper LDL cholesterolemia), but also to select the additional therapies for further LDL reduction on top of statins.

### Study limitations

The retrospective cross-sectional observational study design is a key limitation of our study. Secondly, we excluded subjects for whom relevant clinical and genetic data was not available; this may have introduced an element of selection bias. In addition, there may be a selection bias, where genetic tests were more strongly recommended in the patients with extreme phenotypes or family history. Thirdly, we did not evaluate serum sterol levels which could reassure the pathogenicity of the variants identified in the current study. However, all of the variants in this study have been shown as loss-of function variants leading to cause sitosterolemia [19–21, 24]. Fourthly, we did not observe a significant difference between baseline LDL cholesterol level in mutant group and that in non-mutant group. In this regard, we found that the LDL cholesterol level of mutant group was elevated substantially via *ABCG5* or *ABCG8* genetic variations; on the other hand, that of non-mutant group was elevated to some extent via accumulations of common genetic variations, although we could not account for other environmental factors.

## Conclusion

In summary, our findings revealed that ezetimibe-atorvastatin combination therapy might be more beneficial in hypercholesterolemic patients with a mutation in *ABCG5* or *ABCG8* gene. Genetic analyses on those genes could be useful not only to screening for sitosterolemia, but also to identify individuals with higher responsiveness to ezetimibe.

## Data Availability

The datasets during and/or analyzed during the current study are available from the corresponding author on reasonable request.

## Abbreviations

ABCG5: ATP-binding cassette sub-family G member 5
ABCG8: ATP-binding cassette sub-family G member 8
ASCVD: Atherosclerotic cardiovascular disease
CETP: Cholesteryl ester transfer protein
NPC1L1: Niemann-Pick C1-Like 1
PCSK9: Proprotein convertase subtilisin/kexin type 9
RCT: Randomized controlled trials
SD: Standard deviation
IQR: Interquartile range
